# Do social influences, awareness, or experience matter? Toward a better understanding of Farm-related Injury Risk Perception among agricultural science college students in Ireland

**DOI:** 10.3389/fpubh.2023.1076332

**Published:** 2023-02-27

**Authors:** Mohammad Mohammadrezaei, David Meredith, John McNamara, James Kinsella, Sinéad Flannery

**Affiliations:** ^1^Teagasc – Irish Agriculture and Food Development Authority, Rural Economy Development Programme, Dublin, Ireland; ^2^Teagasc - Irish Agriculture and Food Development Authority, Farm Health and Safety Knowledge Transfer Unit, Kildalton Agricultural College, Kilkenny, Ireland; ^3^School of Agriculture and Food Science, University College Dublin, Belfield, Dublin, Ireland

**Keywords:** Farm-related Injury Risk Perception, experience, awareness, social influences, youth, students, Ireland

## Abstract

**Introduction:**

Formal farm safety education/training should be tailored, in terms of the approach, content and delivery, to students undertaking agriculture education and training to enhance Farm-related Injury Risk Perception (FIRP). To this end, this paper assesses factor(s) explaining or predicting levels of FIRP amongst students studying for a degree in agriculture science.

**Methods:**

A cross-sectional online survey was conducted with a nationally representative sample of Bachelor of Agriculture Science (BAgrSc) students (*N* = 417) (aged 18–20) in Ireland. Descriptive [frequency and cross-tabulations) and inferential (Ordinal Logistic Regression (OLR)] analyses were applied to evaluate the effects of social influences, experience (of farming, of a near-miss or injury), and awareness (of others who were injured or killed on the farm) on FIRP.

**Results:**

The study found that social influences negatively affected FIRP (*P* < 0.05). A relatively small number of students reported experiencing an injury (*n* = 56, 13.4%) that resulted in them being unable to participate in educational or social activities. A quarter of the respondents did, however, record experiencing a near-miss/close call (*n* = 106, 25.4%). A notable proportion (*n* = 144, 34.5%) of students had personal connections to someone who died as a consequence of a farm-related incident and 56.4% (*n* = 235) knew someone who was moderately or severely injured. OLR findings established that experiencing a severe injury, having a near-miss or close call, and awareness of a farm-related death or injury positively affects FIRP (*P* < 0.05).

**Conclusions:**

Perception of farm risks amongst students in Ireland is low. Students who recorded higher levels of risk perception were, however, more likely to report experiencing a near-miss, close call or severe injury, or knowing someone who experienced a farm-related injury or fatality. Farmers, family or friends were found to negatively impact the FIRP and this reflects previous research findings. Our findings highlight the need for education and training programs to enhance opportunities for student peer-to-peer learning through sharing of experiences and/or knowledge of farm injuries and/or fatalities. Such activities will enhance awareness and understanding amongst the general population of students leading to increased FIRP and contribute to a reduction in risk-taking.

## 1. Introduction

Farmers may conscientiously avoid risks if they perceive a higher chance of injury associated with a practice, situation or behavior (1–4). As such, Farm Injury Risk Perception (FIRP) is a key factor which modulates risk taking behavior (5–7) that is distinct to the awareness of the risk, i.e., a person may be aware of a risk yet still engage in a behavior or practice that heightens the possibility of experiencing an injury because they believe themselves to be safe (2, 4, 7). For instance, a farmer may identify dangers associated with animal or tractor/machinery related tasks while perceiving a lower chance of an incidence occurring because they are familiar with the livestock or are experienced operating the machinery (optimistic FIRP) (2, 8–10). In addition to the individual's experience, risk perception and tolerance of risk is also influenced by the social context, i.e., attitudes of family, friends and peers, and the organizational context, i.e., expected behaviors or norms (11–14).

In family farm settings, young adults are one of the main vulnerable groups for farm injury (15, 16). This is explained by a combination of factors including the tendency for the residence and workplace to be in close proximity, a tendency for youth and young adults to work on the farm and take risks (17, 18), and the acceptance of risks amongst farm families (19, 20). Whilst regulatory and educational approaches to increase safety on farms do consider a variety of populations, young adults are generally not the primary focus of such interventions (16, 21–28). An assessment of non-fatal farm incident data indicates that this cohort experiences high levels of injuries or close calls, something that is attributed to higher levels of risk taking (16, 28–34). These data point to the need for FHS initiatives and educational programs targeting young farmers to improve or increase risk perception. A key barrier to the development of education and training courses targeting this population is the absence of research into their appreciation or understanding of key risks they may encounter whilst living or working on a farm. To inform the development of these interventions there is a need for a better understanding of factor(s) explaining or predicting levels of FIRP. Currently, the level of FIRP and the contributory factors among young farmers is something of a “black box” as there is limited research on FIRP (3, 35–37). Accordingly, this paper seeks to identify the predictors (individual, social influences, awareness, and experience) of FIRP amongst a nationally representative population of young adults participating in a BAgrSc course and develop recommendations informing the content and delivery of effective farm safety education and training. Before presenting the approach (methods and data) and results, we map out the conceptual links between FIRP and farmer experience, social influences, and awareness of injuries/fatalities below and specify three core hypotheses. These guide the data collection, choice of methods, analysis and interpretation of results that are reported in the remainder of the paper.

### 1.1. Theoretical framework and hypotheses

#### 1.1.1. Farming experience

According to the literature, to explain the relationship between “farming experiences” and FIRP, in particular among young farmers, it is necessary to understand the extent to which farming experience matters. Within the literature, the role of experience has been reported to have negative and positive influences on risk perception. One body of research finds that experienced farmers with higher working skills and managing/undertaking multiple farming tasks, may have a better judgement of the chance of injury associated with an activity and consequently be more risk averse (35, 38, 39). Another body of research presents evidence indicating experienced farmers may have lower FIRP due to a belief that they are more skilled and capable of controlling risks (4, 9, 40). As such, farming experience might be either positively or negatively associated with the FIRP.

Among young farmers, the same findings have been reported. Limited farming experience is one of the factors associated with the underestimation of risks by young farmers (2, 35). New entrants to farming, even those from a farming background, have been found to have a relatively low appreciation of the occupational risks they face (19). Notwithstanding this, growing up on farms and having greater farming experience may not necessarily be positive as it can contribute to maladaptive behaviors or overconfidence (14, 36). It is also acknowledged that as younger farmers gain experience or age, they are given more responsibility for additional tasks. Whilst these tasks may be riskier in and of themselves, the increased workload and greater time working, frequently on their own, represents risks (9, 14, 41, 42).

Based on the current literature it is uncertain how or to what extent experience of farming shapes the FIRP of young farmers and this informs our first hypothesis:

H1a: Farming experience is positively associated with higher levels of FIRP among young farmers.

#### 1.1.2. Experience of injury or near miss/close call and injury experience

Apart from general farming experience, the experience of a farm-related incident or injury including a near miss or close call is considered a key factor contributing to FIRP and, consequently, risk prevention behavior (7, 9, 36, 43). A number of studies have found that farmers who experienced a near miss were more risk adverse (44–46). Similarly, being injured as a consequence of risk taking behavior may positively shape FIRP as farmers learn from the experience (4, 9, 28, 43, 47). There is some evidence that the positive impact a recent experience of a near miss/close call may have on FIPR can weaken over time (9).

In contrast to this body of research, other studies have highlighted that experiencing a near miss or close call may negatively influence FIRP (44–46, 48) as individuals perceive a lower chance of severe injury and a higher level of control over such situations in the future. It is also argued that farmers may take additional risks if they experienced a minor injury or near miss/close call (9, 42). When it comes to younger farmers, this factor might be even more important as this population are less likely to experience severe injury and more likely to experience a near miss/ close call which may negatively affect the level of FIRP (16, 32–34). There is, however, no clear evidence regarding the impact of experiencing a near miss or close call on the risk perception of younger farmers. This informs the development of two related hypotheses which are tested below:

H1b: The experience of a near miss or close call will positively affect FIRP among young farmers.
H1c: The experience of a severe farm-related injury will positively affect FIRP among young farmers.

#### 1.1.3. Social influences

In addition to individual experience of farming, social influences are recognized as playing a significant role in the formation of FIRP (49, 50). Culturally, risk-taking may be considered a “social value” and thus is accepted and normalized as “what a farmer is expected to do” (19, 42, 51). Some research implies that FIRP is built up through social confirmation or pressure from key people or referents named as “important others” both within the household and the wider social or community network (13, 20, 52). Young farmers are particularly influenced by family members including parents, grandparents, uncles, aunts etc. (36, 39, 41, 50). This influence works in two ways, firstly through transmission of views on what constitutes a risk and the levels of ‘acceptable' risk associated with different practices (2, 13, 36, 52), and secondly, social pressure to adopt risky behaviors (4, 20, 49, 53), e.g., operating machinery or working with livestock without adequate training or protective equipment/facilities which are commonly perceived to result in higher economic returns (2, 9, 14, 36). Engaging in risky activities is also seen as a way of accruing cultural capital amongst peers, including other farmers and friends, i.e., demonstrating that they are “authentic” farmers (4, 19, 38). Counterbalancing these potentially negative influences, young farmers are also exposed to a range of other social influences, e.g., lecturers or farm advisors who hold positions of authority and, consequently, may be in a strong position to positively influence FIRP. In order to assess the effect of the variety of social influences on FIRP this study examines the following hypotheses:

H2a: Social influences from (a) family/friends and (b) farmers will negatively affect FIRP among young farmers.
H2b: Social influences from lectures/advisors (c) will positively affect FIRP among young farmers.

#### 1.1.4. Awareness of farm related injury/fatal incidents

Farmers who know someone who died or was injured as a consequence of a farm incident are more risk averse than the general population of farmers (13, 14, 31, 44). Notwithstanding this, there is a knowledge deficit in understanding to what extent hearing about or knowing someone who has been injured/killed as a result of an incident affects FIRP, particularly among young farmers. Accordingly, the following hypothesis is examined in this study:

H3: Knowing someone who was: (a) injured; or (b) killed, resulting from a farm-related incident positively affects FIRP among young farmers.

## 2. Materials and methods

### 2.1. Participants

To test the study hypotheses, this study applied a cross-sectional quantitative approach. First and second year undergraduate students enrolled in agriculture related education courses at the School of Agriculture and Food Science in University College Dublin (UCD), formed the target population. This population was selected as these students generally come from farm families, though not in every case, and generally go on to work as farmers or within the wider agriculture sector that supports farm businesses e.g., farm extension services, financial services, or agri-food businesses (54). The exclusion of third or fourth year students avoided a potential effect/bias as these have to complete a “Health, Welfare, and Safety” module prior to engaging in professional work experience placements in third year.

### 2.2. Procedure

To recruit a representative sample of this population, according to Kerjcy and Morgan's (55) sampling equation,


(1) $$\begin{array}{l} S=X^{2}NP\left(1-P\right)/\;d^{2}\;\left(N-1\right)+\;X^{2}P\left(1-P\right) \end{array}$$

Where S is the required sample size, *X* equates to *Z* value (1.96 for 95% confidence level), *N* refers to the population size which is all Bachelor of Agricultural Science (BAgrSc) students (*N* = 545), and P is the population proportion (considered as a decimal) (assumed to be 0.5 (50%), and d is margin of error (5%), (expressed as a proportion (0.05)). This provides an estimate of a minimum of 148 students needed as the sample size (95% confidence level). To access this population we distributed the survey using the online Survey Monkey platform to lecturers who, in turn, raised awareness of the survey amongst the target population. The authors sought ethical exemption, which was granted and a letter of permission from the Head of School provided. In applying for the exemption, the authors set out the potential ethical issues relating to the proposed research and the protocols that were put in place. The design of the survey reflected this and took into consideration the potential that some questions, particularly those that asked about knowledge or experience of serious farm injuries or fatalities, had the potential to cause upset or anxiety to respondents. In anticipating this, prior to respondents answering any questions, we provided them with an outline of the types of questions and the issues covered in the survey, highlighted that they did not have to participate, could withdraw from the survey at any stage and could request that their data be removed from the analysis at any time. The anonymity of the participants was assured by not collecting any information that could be used to identify individuals. Finally, lecturers were asked to highlight resources and student support services available to students in the university. In total, 417 students completed the survey (Response rate = 76.5%) which was greater than the expected response rate for online surveys (33–47%) (56) and above the minimum sample required.

### 2.3. Measures

The survey comprised a number of distinct sections that are outlined below.

#### 2.3.1. Demographic variables

Demographic variables included age, stage of university education (i.e., first or second year of study), and gender (male/female/prefer not to say).

#### 2.3.2. Dependent and independent variables

##### 2.3.2.1. FIRP (dependent variable)

FIRP may vary depending on different activities (7, 10). As a consequence FIRP was measured by four questions that assessed “general perception,” questions 1 and 2, and perception of “specific” risks, questions 3 and 4, related to machinery and livestock. These specific risks correspond to the primary causes of fatal farm injuries in Ireland (15) and many other countries (57, 58),

1. *How likely or unlikely do you think it is that you may get injured whilst working on the farm? (FIRP1)*
2. *How likely or unlikely do you think it is that a family member may get injured on the farm? (FIRP2)*
3. *How likely or unlikely do you think it is that you may get injured whilst working with farm machinery, e.g., tractor, harvesting machines, quad etc.? (FIRP3)*
4. *How likely or unlikely do you think it is that you may get injured whilst working with livestock? (FIRP4)*

All items were measured using a 5-point Likert scale ranging from 1 “very unlikely” to 5 “very likely.” Responses were categorized into one of three groups; “low FIRP” where respondents selected “very unlikely or unlikely”; medium if they selected “neutral FIRP”; or “high FIRP” if they chose “likely or very likely.”

##### 2.3.2.2. Independent variables

The independent variables consisted of estimates of the level of farming experience with response options ranging from “none” though to “a great deal.” Experience of a near miss or close call was measured through two binary questions. Experience of an injury was assessed through a single binary question that asked if the respondent had been unable to participate in education or work activities for more than a day as a consequence of having a work related injury in the preceding 12 months. With regard to social influences, respondents were asked to rank in order of importance those who influence their decisions regarding farm safety issues. Response options included “family/friends, “farmers,” “farm advisors/lecturer” and others. Subsequent questions sought to explore knowledge of someone who experienced a severe farm-related injury or fatality. We used two separate binary questions to assess knowing someone who “died” and knowing someone who was “severely injured.”

### 2.4. The questionnaire validity and reliability

To validate the degree to which each variable is accurately measured by items/questions, the questionnaire was tested to ensure content and face validity. Following Engel et al. (59) recommendations, the identification of potential dependent and independent variables from the literature review preceded an evaluation of these by a panel of experts/researchers (59). The panel included three FHS specialists along with four behavioral and social scientists with a previous background in FHS studies. The content of each variable was reviewed and their validity assessed (59). The panel evaluated the face validity of each question, i.e., the degree to which questions measuring study variables were relevant to the target population (60). Questions were evaluated based on “feasibility, readability, consistency of style and formatting, and the clarity of the language used” (60).

To assess the face validity and reliability of the questionnaire items, a pilot study was undertaken among 54 college students that were participating in agricultural education. As there was no construct containing multiple ordinal/binary items to compute, a reliability test was not needed. However, this paper estimated Cronbach's alpha for FIRP (four items) (0.89) which was greater than the acceptable cut-off point (>0.7) (61).

During this phase of the research consideration was given to how to avoid optimistic or social desirability bias. A number of strategies were identified and implemented, including assuring students of their anonymity, and providing a brief overview of the study objectives that stressed the importance of factual, rather than desired, responses (62).

### 2.5. Statistics

#### 2.5.1. Descriptive analysis

In the first step, frequencies and cross-tabulation analysis were conducted to describe the level of FIRP and distribution relative to the demographic and independent variables. Cross-tabulation analyses using Kendal tau b and c were applied to examine the correlations between both demographic and independent factors with FIRP. All variables which were found to be significantly associated with FIRP were entered into the regression model.

#### 2.5.2. Ordinal Logistic Regression

FIRP comprises three ordinal categories, “low,” “neutral,” and “high.” To assess the relationship with the independent variables, an Ordinal Logistic Regression (ORL) technique is applied. As the ordinal FIRP categories of “low,” “neutral,” and “high” were computed from five point Likert scales, the proportional odds model was applied (63). This technique explains the effects of exogenous nominal and ordinal variables on FIRP as an ordinal (dependent) variable by one regression coefficient (63). Accordingly, the regression findings are relatively easy to convey to a wider range of stakeholders who may not be familiar with this type of analysis (63).

Prior to testing the study hypotheses, the goodness of fit of the regression model was estimated (63). As the FIRP was an ordinal categorical variable with a limited number of categories (three), the Person chi-square statistic was applied to estimate the global goodness of fit of the regression model (63). To perform the analyses, SPSS for Windows (Version 27.0. Armonk, NY) (64) was used.

## 3. Results

### 3.1. Descriptive statistics

#### 3.1.1. Characteristics of respondents

In terms of the sample, 45.8% (*n* = 191) and 54.2% (*n* = 226) were in first and second years of their BAgrSc, respectively. The median age of respondents was 19 years (mean = 18.91 ± 0.79). Just over half of respondents were female (*n* = 222, 53.2%). Describing the farming experience, over half of the students reported their level of engagement in farming activities as “A great deal” (*n* = 222, 53.2%), followed “quite a bit” (*n* = 80, 19.2%). In turn, students indicated their farming experience as “Some” (*n* = 46, 11 %), “A little” (*n* = 35, 8.4%), and “None” (*n* = 34, 8.2%). As such, the majority of students are substantially engaged in farming activities (over 70%), and can be considered to be “young farmers.”

One quarter (*N* = 106, 25.4%) of respondents reported experiencing a near-miss or close call over the previous 12 months. Of this number, 52 (49.06%) reported the primary cause of the incident involved livestock, 26 (24.52%) involved a tractor, 23 (21.67%) involved farm machinery, and 5 (4.21%) were recorded as “other.” Only 58 (13.9%) students reported experiencing severe farm-related injuries resulting in being unable to participate in education or work activities for more than a day over the previous 12 months. Livestock (*n* = 21, 36.2%), farm machinery (*n* = 18, 31.03%), and tractors (13, 22.41%) were the three main causes of such incidents. Regarding students' awareness of farm injury/fatal incidents, more than half of the participants (*n* = 235, 56.4%) identified that they know someone who was injured as a result of a farm-related incident. Furthermore, 144 students (34.5%) reported that they know someone who died as a result of farm-related incidents. “Farmers” were identified as the key “important others” by nearly one-third of respondents (*n* = 161, 38.6%), followed by “family/friends” (*n* = 140, 33.6%). Finally, just 57 students (13.7%) identified “farm advisors/university lecturers” as the key “important others.” Accordingly, farmers and family/friends are the key “important others” whose thoughts/views on FHS matter most to students.

#### 3.1.2. Farm-related Injury Risk Perception

**Figure 1:**
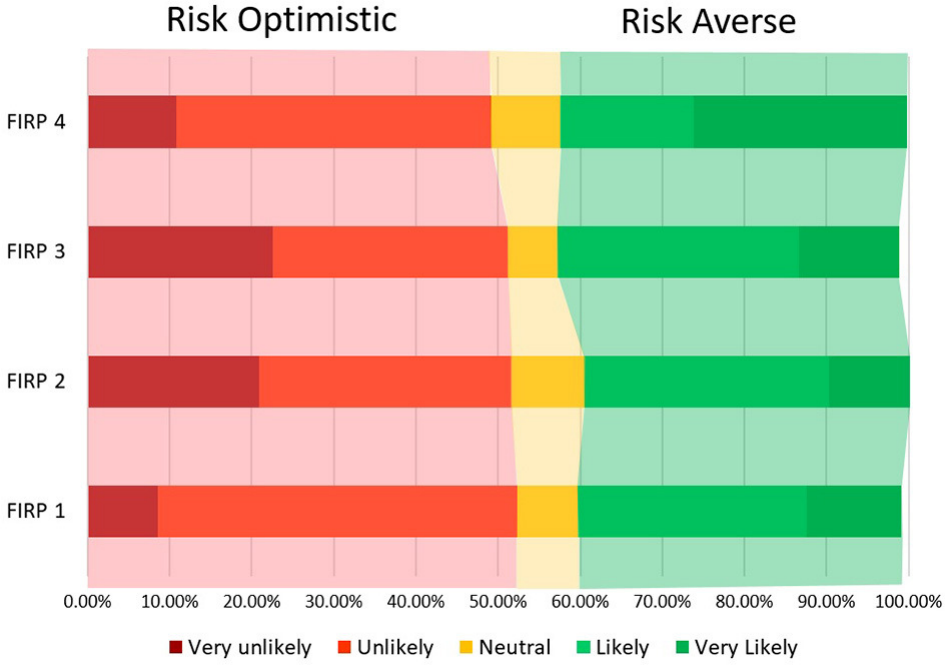
The level of the four FIRP dimensions.

The responses of students to the FIRP questions were assessed and two distinct groups, those who are risk optimistic, i.e., they perceive lower levels of risk, and those who perceive higher levels of risk and are more risk averse. The absence of an intermediate group, i.e. those who are uncertain, suggests that risk perception is a construct with little room for ambiguity. Assessing the four measures used to assess FIRP, we find that the pattern of responses are broadly similar with, roughly 50–52% of respondents reporting lower levels of risk perception, 40–42% reporting higher levels, whilst 8%, on average, have neutral perceptions (Figure 1).

### 3.2. Correlations

#### 3.2.1. Background factors

Before analyzing the correlations between independent variables (experience, social influences, and awareness) and the four FIRP dimensions, this study examined if there are significant correlations between age, year of education, and FIRPs to identify and if necessary, control for any biases from these factors on the FIRPs. Regarding age, which was non-normally distributed (most students are the same age, mean = 18.91 ± 0.79), we used Kruskal-Wallis H Test to examine if there are any differences between the age of students who identified FIRPs to be “low,” “neutral,” and “high” and found that there is no significant difference between the age of students across the three risk cohorts and the four FIRP dimensions (*P* > 0.05). Therefore, we conclude that, with regards this sample, age was not a factor associated with students' judgement on the likelihood of farm-related injury incidents.

#### 3.2.2. Farming experience

There was no significant association between “farming experience” and students' level of risk perception (*P* = 0.16–0.36). As such this finding leads us to reject H1a, i.e., that farming experience is positively associated with higher levels of FIRP among young farmers. Cross-tabulation analysis revealed that students with higher farming experience (“a great deal” and “quite a bit”) are almost equally likely to be “risk optimistic” (ranging from 46.4 to 57.2%) or “risk averse” (37.9–44.1%) across all four FIRP dimensions (Tables 1–4). The same pattern was identified for students with most of the other levels of experience (Tables 1–4). The one group that stood out were those students who classified themselves as having “some” farming experience. Here we found that, depending on the FIRP, between 54.3 and 67.4% perceived lower risk of farm-related injury incidents, i.e., they were, in general, more optimistic than their counterparts. We found that students with “none” or “a little” farming experience are, in general, “risk optimistic” as over half of them perceived lower FIRPs.

**Table 1:** Chi-square analysis between background and explanatory variables and FIRP1.

**Variables**		**FIRP1 (likelihood of farm related injury occurrence while working on farm in general)**						**Stats**
		**Low (218, 52.3%)**		**Neutral (31, 7.4%)**		**High (168, 40.3%)**		**Test**
		***n***	**%**	***n***	**%**	***n***	**%**	
Background variables	**Year of study**								
	First stage (191, 45.8%)	100	52.4	14	7.3	77	40.3	2.054
	Second stage (226, 54.2%)	118	52.2	17	7.5	91	40.3	
	**Gender**								
	Male (195, 46.8%)	103	52.8	16	8.2	76	39	0.47
	Female (222, 53.2%)	115	51.8	15	6.8	92	41.4	
	**Farming experience**								
	A great deal (222, 53.2%)	111	50	18	8.1	93	41.9	13.49
	Quite a bit (80, 19.2%)	40	50	6	7.5	34	42.5	
	Some (46, 11%)	31	67.4	4	8.7	11	23.9	
	A little (35, 8.4%)	23	65.7	2	5.7	10	28.6	
	None (34, 8.2%)	13	38.2	1	2.9	20	58.8	
Social influences	**a. Family/friends**								
	Yes (140, 33.6%)	94	67.1	12	8.6	34	23.6	6.35^*^
	No (277, 66.4%)	144	52.0	19	6.9	114	41.2	
	**b. Farmers**								
	Yes (161, 38.6%)	110	68.3	7	4.3	44	27.3	27.2^***^
	No (256, 61.4%)	108	42.2	24	9.4	124	48.4	
	**c. Advisors/lecturers**								
	Yes (57, 13.7%)	15	26.3	23	40.4	39	34.5	1.92^***^
	No (360, 86.3%)	203	56.4	28	7.8	129	35.8	
Experience	**Near miss/close call**								
	Yes (106, 25.4%)	9	8.5	1	0.9	96	90.6	149.4^***^
	No (311, 74.6%)	209	67.2	30	9.6	72	23.2	
	**Severe injury**								
	Yes (58, 13.9%)	1	0	0.0	0.0	57	98.3	94.17^***^
	No (359, 86.1%)	217	60.4	31	8.6	111	30.9	
Awareness	**Someone who injured**								
	Yes (235, 56.4%)	91	38.7	14	6.0	130	55.3	50.69^***^
	No (182, 43.6%)	127	69.8	17	9.3	38	20.9	
	**Someone who died**								
	Yes (144, 34.5%)	21	14.6	10	6.9	113	78.5	139^***^
	No (273, 65.5%)	197	72.2	21	7.7	55	20.1	

* *P* < 0.05;

^**^P < 0.01;

*** *P* < 0.001.

**Table 2:** Chi-square analysis between background and explanatory variables and FIRP2.

**Variables**		**FIRP2 (likelihood of farm related injury occurrence to family members while working and living on farm)**						**Stats**
		**Low (215, 51.6%)**		**Neutral (37, 8.9%)**		**High (165, 39.6%)**		**Test**
		***n***	**%**	***n***	**%**	***n***	**%**	
Background variables	**Year of study**								
	First stage (191, 45.8%)	94	49.2	21	11	76	39.8	2.16
	Second stage (226, 54.2%)	121	53.5	16	7.1	89	39.4	
	**Gender**								
	Male (195, 46.8%)	101	51.8	19	9.7	75	38.5	0.43
	Female (222, 53.2%)	114	51.4	18	8.1	90	40.5	
	**Farming experience**								
	A great deal (222, 53.2%)	106	47.7	24	10.8	92	41.4	12.18
	Quite a bit (80, 19.2%)	40	50	6	7.5	34	42.5	
	Some (46, 11%)	25	54.3	4	8.7	17	37	
	A little (35, 8.4%)	23	65.7	2	5.7	10	28.6	
	None (34, 8.2%)	17	50	1	2.9	16	47.1	
Social influences	**a. Family/friends**								
	Yes (140, 33.6%)	93	66.42	17	12.1	30	21.43	6.48^**^
	No (277, 66.4%)	146	52.7	20	7.2	111	40.1	
	**b. Farmers**								
	Yes (161, 38.6%)	112	69.6	8	5.0	41	25.5	
	No (256, 61.4%)	103	40.2	29	11.3	124	48.4	34.18^***^
	**c. Advisors/lecturers**								
	Yes (57, 13.7%)	28	49.12	3	5.3	26	45.6	2.3
	No (360, 86.3%)	203	56.4	28	7.8	129	35.8	
Experience	**Near miss/close call**								
	Yes (106, 25.4%)	9	8.5	1	0.9	96	90.6	149.4^***^
	No (311, 74.6%)	206	66.2	36	11.6	69	22.2	
	**Severe injury**								
	Yes (58, 13.9%)	1	0	0.0	0.0	57	98.3	97.11^***^
	No (359, 86.1%)	214	59.6	37	10.3	108	30.1	
Awareness	**Someone who injured**								
	Yes (235, 56.4%)	90	38.3	17	7.2	128	54.5	50.20^***^
	No (182, 43.6%)	125	68.7	20	11	37	20.3	
	**Someone who died**								
	Yes (144, 34.5%)	20	13.9	9	6.3	115	79.9	152.5^***^
	No (273, 65.5%)	195	71.4	28	10.3	50	18.3	

^*^P < 0.05;

** *P* < 0.01;

*** *P* < 0.001.

**Table 3:** Chi-square analysis between background and explanatory variables and FIRP3.

**Variables**		**FIRP3 (likelihood of farm related injury occurrence whilst working with farm machinery, e.g., tractor, harvesting machines, quad etc.)**						**Stats**
		**Low (218, 52.3%)**		**Neutral (25, 6%)**		**High (174, 91.7%)**		**Test**
		***n***	**%**	***n***	**%**	***n***	**%**	
Background variables	**Year of study**								
	First stage (191, 45.8%)	97	50.8	14	7.3	80	41.9	1.19
	Second stage (226, 54.2%)	121	53.5	11	4.9	94	41.6	
	**Gender**								
	Male (195, 46.8%)	111	56.9	10	5.1	74	37.9	0.29
	Female (222, 53.2%)	123	55.4	14	6.3	85	38.3	
	**Farming experience**								
	A great deal (222, 53.2%)	127	57.2	7	3.15	88	39.6	12.18
	Quite a bit (80, 19.2%)	41	51.2	5	6.3	34	42.5	
	Some (46, 11%)	28	60.9	3	6.5	15	32.6	
	A little (35, 8.4%)	23	65.7	2	5.7	10	28.6	
	None (34, 8.2%)	15	44.1	0	0	19	55.9	
Social influences	**a. Family/friends**								
	Yes (140, 33.6%)	90	64.3	11	7.9	39	27.9	9.95^**^
	No (277, 66.4%)	144	52	13	4.7	120	43.3	
	**b. Farmers**								
	Yes (161, 38.6%)	106	65.8	13	8.1	42	26.1	16.22^**^
	No (256, 61.4%)	100	39.1	22	8.6	134	52.3	
	**c. Advisors/lecturers**								
	Yes (57, 13.7%)	23	40.4	6	10.5	28	49.1	1.36
	No (360, 86.3%)	221	61.4	23	6.4	116	32.2	
Experience	**Near miss/close call**								
	Yes (106, 25.4%)	9	8.5	2	1.9	95	90.6	131.03^***^
	No (311, 74.6%)	197	63.3	33	10.6	81	22.2	
	**Severe injury**								
	Yes (58, 13.9%)	1	1.7	0.0	0.0	57	98.3	97.11^**^
	No (359, 86.1%)	205	57.1	3	9.7	119	33.1	
Awareness	**Someone who injured**								
	Yes (235, 56.4%)	82	34.9	18	7.7	135	57.4	52.92^**^
	No (182, 43.6%)	124	68.1	17	9.3	41	22.5	
	**Someone who died**								
	Yes (144, 34.5%)	21	14.6	11	7.6	112	77.8	141.2^***^
	No (273, 65.5%)	200	73.3	13	4.8	60	22.0	

^*^P < 0.05;

** *P* < 0.01;

*** *P* < 0.001.

**Table 4:** Chi-square analysis between background and explanatory variables and FIRP4.

**Variables**		**FIRP4 (likelihood of farm related injury occurrence while working with livestock)**						**Stats**
		**Low (206, 49.4%)**		**Neutral (35, 8.4%)**		**High (176, 42.2%)**		**Test**
		***n***	**%**	***n***	**%**	***n***	**%**	
Background variables	**Year of study**								
	First stage (191, 45.8%)	94	48.2	18	9.2	83	42.6	2.05
	Second stage (226, 54.2%)	112	50.5	17	7.7	93	41.9	
	**Gender**								
	Male (195, 46.8%)	111	56.9	10	5.1	74	37.9	0.42
	Female (222, 53.2%)	123	55.4	14	6.3	85	38.3	
	**Farming experience**								
	A great deal (222, 53.2%)	103	46.4	21	9.5	98	44.1	11.49
	Quite a bit (80, 19.2%)	39	48.8	6	7.5	35	43.8	
	Some (46, 11%)	28	60.9	3	6.5	15	32.6	
	A little (35, 8.4%)	24	68.6	2	9	9	25.7	
	None (34, 8.2%)	12	35.3	3	8.8	19	55.9	
Social influences	**a. Family/friends**								
	Yes (140, 33.6%)	85	60.71	10	7.14	45	32.14	18.35^***^
	No (277, 66.4%)	138	49.8	22	7.9	117	42.2	
	**b. Farmers**								
	Yes (161, 38.6%)	106	65.8	13	8.1	42	26.1	30.52^***^
	No (256, 61.4%)	100	39.1	22	8.6	134	52.3	
	**c. Advisors/lecturers**								
	Yes (57, 13.7%)	31	54.4	6	10.5	20	35.1	1.23
	No (360, 86.3%)	221	61.4	23	6.4	116	32.2	
Experience	**Near miss/close call**								
	Yes (106, 25.4%)	22	20.8	1	0.9	83	78.3	97.54^***^
	No (311, 74.6%)	212	68.2	23	7.4	76	24.4	
	**Severe injury**								
	Yes (58, 13.9%)	1	1.7	0.0	0.0	57	98.3	61.62^***^
	No (359, 86.1%)	205	57.1	3	9.7	119	33.1	
Awareness	**Someone who injured**								
	Yes (235, 56.4%)	100	42.6	15	6.4	120	51.1	41.64^***^
	No (182, 43.6%)	134	73.6	9	4.9	39	21.4	
	**Someone who died**								
	Yes (144, 34.5%)	34	23.6	11	7.6	99	68.8	138.3^***^
	No (273, 65.5%)	188	68.9	26	9.5	59	21.6	

^*^P < 0.05;

^**^P < 0.01;

*** *P* < 0.001.

#### 3.2.3. Social influences

There was a statistically significant association between respondents who reported family/friends (*P* = 0.001–0.036) or farmers (*P* = 0.001) as the key “important others” and the level of each of the four FIRPs (Tables 1–4). We found students who are influenced by their family/friends are more likely to be “risk optimistic.” The analysis establishes that the majority of this cohort (60.71–67.1) perceived a “low” level of FIRPs. This is similar to the proportion (ranging from 65.8% to 69.6% depending on the FIRP) of respondents who reported “farmers” as being a key influence (Tables 1–4). There was no significant association between students who reported “advisors/university lecturers” as the key social referents and the levels of FIRPs. These results lead us to accept H2a, i.e., that social influences from family/friends (a) and farmers (b), will negatively affect FIRP among young farmers, and reject H2b, i.e., that social influences from lectures/advisors (c) will positively affect FIRP among young farmers.

#### 3.2.4. Experience of near miss/close call or severe injury

Experience of a “near miss/close call” (*P* = 0.001), and experiencing a “severe farm-related injury” (*P* = 0.001) were statistically significantly associated with the level of FIRPs leading us to accept H1b (Tables 1–4). Almost all students, *n* = 106 (25.4%), who have experienced a “near miss/close call” in the past year reported higher levels of FIRPs, i.e., ranging between 78.3% for livestock to 90.6% for general risks to self. Similarly, almost all students (*n* = 58) who have experience of a severe farm-related injury reported significantly (*P* = 0.001) higher FIRPs compared to others (Tables 1–4) leading us to accept H1c. In each FIRP, 98.3% of respondents reported “higher” risk perception. This result is interesting in that it suggests experiencing a severe injury impacts both general and specific risk perception. This contrasts with having a close call which has a more variable impact on, particularly, specific risk perception.

#### 3.2.5. Awareness of fatal and non-fatal incidents

The data establishes that 235 (56.4%) students know of someone who experienced a severe farm incident whilst 144 (34.5%) know of someone who was killed as a consequence of a fatal farm incident. In both instances, awareness of fatal and non-fatal incidents positively, and significantly, impacts on all FIRPs leading us to accept H3. Students who know someone who was severely injured or killed are more risk averse compared to others (*P* = 0.001). Accordingly, awareness of fatal incidents and farm-related injuries are clearly associated with the level of FIRPs (Tables 1–4).

### 3.3. OLR analysis

To further examine the study hypotheses four OLRs were developed to estimate the causal effects of social influences of “family/friends” and “farmers,” the experience of a “near miss/close call” and “severe injury,” and awareness of “fatal farm incidents” and “farm injury incidents” on each FIRP. Variables describing “general farming experience” and social influences of “advisors/lecturer,” which were not statistically significantly associated with the four dimensions of FIRP, were excluded from the models. The goodness of fit index (chi-square) for each model was found to be statistically significant (*P* = 0.001) indicating four models fits this set of data (63). The Nagelkerke *R*^2^ value of the four models explained, respectively, 51%, 59%, 56%, and 53% of the total variation associated with each FIRP (Table 5).

**Table 5:** Ordinal Logistic Regression analysis between explanatory variables and FIRPs.

**Independent variables**		**Multivariate model 1, FIRP1**		**Multivariate model 2, FIRP2**		**Multivariate model 3, FIRP3**		**Multivariate model 4, FIRP4**	
		**OR** ^a^	**95%CI** ^b^	**OR** ^a^	**95%CI** ^b^	**OR** ^a^	**95%CI** ^b^	**OR** ^a^	**95%CI** ^b^
Social influences	**Family/friends**								
	Yes	−0.89^*^	(−1.2) to (−0.15)	−0.57^*^	(−1.28) to (0.14)	−0.93^**^	(−1.66) to (−0.2)	−0.81^**^	(−1.52) to (−0.1)
	No	(Indicator variable)		(Indicator variable)		(Indicator variable)		(Indicator variable)	
	**Farmers**								
	Yes	−1.68^***^	(−1.88) to (−0.44)	−1.17^**^	(−1.9) to (0.46)	−1.37^**^	(−1.88) to (−0.46)	−1.34^**^	(−2.04) to (−0.66)
	No	(Indicator variable)		(Indicator variable)		(Indicator variable)		(Indicator variable)	
Experience	**Near miss/close call**								
	Yes	2.61^***^	(1.86) to (3.36)	2.66^***^	(1.9) to (3.4)	2.43^***^	(1.69) to (3.17)	2.26^***^	(1.53) to (2.99)
	No	(Indicator variable)		(Indicator variable)		(Indicator variable)		(Indicator variable)	
	**Severe injury**								
	Yes	2.93^***^	(0.86) to (4.9)	2.74^***^	(0.68) to (4.8)	2.75^***^	(0.7) to (4.8)	2.72^***^	(0.66) to (4.7)
	No	(Indicator variable)		(Indicator variable)		(Indicator variable)		(Indicator variable)	
Awareness	**Knowing someone who injured**								
	Yes	0.53^*^	(0.0) to (1.05)	0.45^*^	(−0.07) to (0.98)	0.48^*^	(0.47) to (1.51)	0.56^**^	(0.05) to (1.07)
	No	(Indicator variable)		(Indicator variable)		(Indicator variable)		(Indicator variable)	
	**Knowing someone who died**								
	Yes	1.81^**^	(1.23) to (2.38)	1.98^***^	(1.41) to (2.57)	1.69^***^	(1.11) to (2.26)	1.89^***^	(1.32) to (2.47)
	No	(Indicator variable)		(Indicator variable)		(Indicator variable)		(Indicator variable)	
	Chi-square (final)	173.191^***^		384.325^***^		261.04^***^		265.15^***^	
	**Pseudo R-square**								
	Nagelkerke	0.51		0.59		0.56		0.53	

* *P* < 0.05;

** *P* < 0.01;

*** *P* < 0.001.

a Odd Ratio.

b Confidence interval at 95%.

Having previously established that experience of a near miss/close call positively and significantly influences the level of FIRPs (H1b), the OLR analysis provides an estimate of the effect. The largest effects are associated with perception of risk to self, FIRP1 [Odd Ratio (OR): 2.61, *P* = 0.001], and others, FIRP2 (OR: 2.66, *P* = 0.001). Slightly smaller effects are associated with livestock, FIRP4 (OR: 2.43, *P* = 0.001), and tractor or machinery risks, FIRP3 (OR: 2.26, *P* = 0.001) (Table 5). Overall we find that students with such experience are roughly two and half times more likely to perceive a “*high*” level of FIRP. This compares to students without this experience who are more likely to perceive a “*low*” level of FIRP across each of the measures.

**Figure 2:**
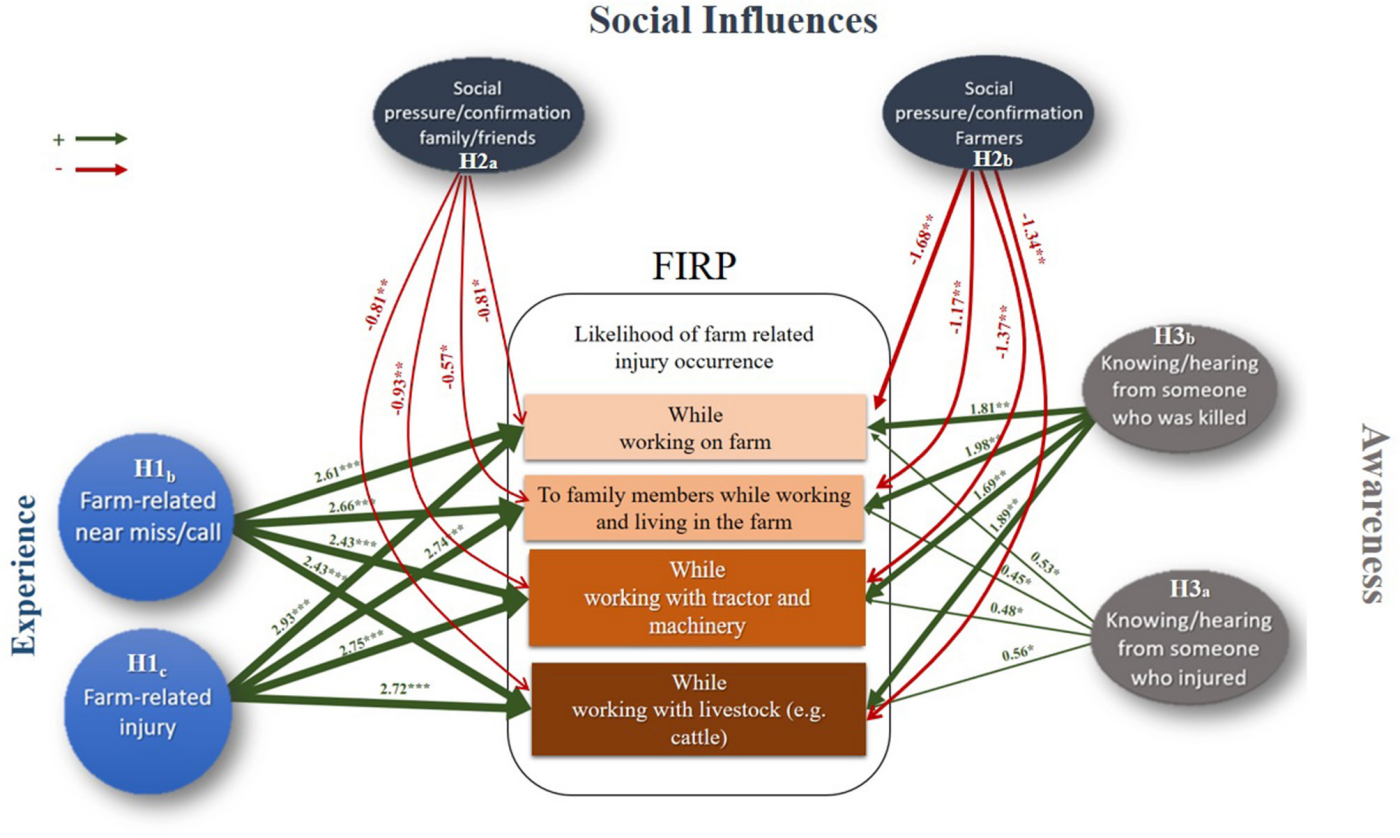
Experimental model of factors affecting FIRP.

Experience of a severe injury in the past year was also shown to positively impact on FIRPs (H1c). Applying OLR we estimate that this cohort of young farmers (58, 13.9%) are almost three times more likely to perceive higher FIRP1 (OR: 2.93, *P* = 0.034), FIRP2 (OR: 2.74, *P* = 0.008), FIRP3 (OR: 2.75, *P* = 0.005), and FIRP4 (OR: 2.72, *P* = 0.009) compared to their counterparts who do not have this experience. Comparing the odd ratios estimated for the experience of “*near miss/close call*” with experiencing a “*severe injury*” it is clear that the latter has a greater impact on risk perception (Table 5, Figure 2).

In contrast with the positive influences of “*experience*,” social influences of family/friends or farmers (H2a) were identified as negative factors influencing the FIRPs. Regarding the social impacts of “*family/friend*,” we found that students who identified this cohort as the key social referent regarding farm safety issues are more likely to underestimate FIRP1 (OR: −0.89, *P* = 0.001), FIRP2 (OR: −0.57, *P* = 0.02), FIRP3 (OR: −0.93 2.72, *P* = 0.01), and FIRP4 (OR: −0.81, *P* < 0.009) and, consequently, are more “*risk optimistic*” (Table 5). Similar to family/friends, the social influences of “*farmers*” as the main “*important others*” is a negative factor which significantly affects FIRP1 (OR: −1.68, *P* = 0.001), FIRP2 (OR: −1.17, *P* = 0.001), FIRP3 (OR: −1.37, *P* = 0.001), and FIRP4 (OR: −1.34, *P* = 0.001). It is evident from these data that the negative influence of “*farmers*” is substantially higher than “*family/friends*.”

Awareness of “*fatal*” and “*non-fatal*” incidents on FIRPs (H3) was found to impact all FIRPs. The OLR analysis estimated the level for those who knew of someone who was injured to be slightly positive; FIRP1 (OR: 0.53, *P* = 0.012), FIRP2 (OR: 0.45, *P* = 0.023), FIRP3 (OR: 0.48, *P* = 0.018), and FIRP4 (OR: 0.56, *P* =0.031) (Table 5). Knowing someone who died in a farm-fatal incident had a much greater effect and positively influences the level of FIRPs (Table 5). The risk perception of students who knew someone who died in a farm incident was over 1.6 higher than that of their counterparts without this knowledge, i.e., FIRP1 (OR: 1.81, *P* = 0.001), FIRP2 (OR: 1.98, *P* = <0.006), FIRP3 (OR: 1.69, *P* = 0.003), FIRP4 (OR: 1.89, *P* = 0.001).

## 4. Discussion

In answer to the overarching question that guides this paper, we found that experience, social influences, and awareness do matter when it comes to shaping young farmers' risk perception. Worryingly but not surprisingly, the analysis leads us to accept H2b, i.e., that social influences of, particularly “*farmers*,” were identified as having the largest and most negative effect on FIRPs. More positively, our experimental model (Figure 2), identifies experiences that increased FIRP, i.e. having a near miss or close call (H1b), experiencing a severe injury (H1c), and knowledge of someone who died in a farm incident (H3b) all have positive effects on risk perception. We provide a summary of these findings in Table 6.

**Table 6:** Summary of hypothesis tests.

**Hypothesis**	**Result**
H1a: Farming experience is positively associated with higher levels of FIRP among young farmers.	Rejected
H1b: The experience of a near miss or close call will positively affect FIRP among young farmers	Accepted
H1c: The experience of a severe farm-related injury will positively affect FIRP among young farmers	Accepted
H2a: Social influences from (a) family/friends and (b) farmers will negatively affect FIRP among young farmers	Accepted
H2b: Social influences from (c) lectures/advisors will positively affect FIRP among young farmers	Rejected
H3: Knowing someone who was: (a) injured; or (b) killed, resulting from a farm-related incident positively affects FIRP among young farmers	Accepted

**Figure 3:**
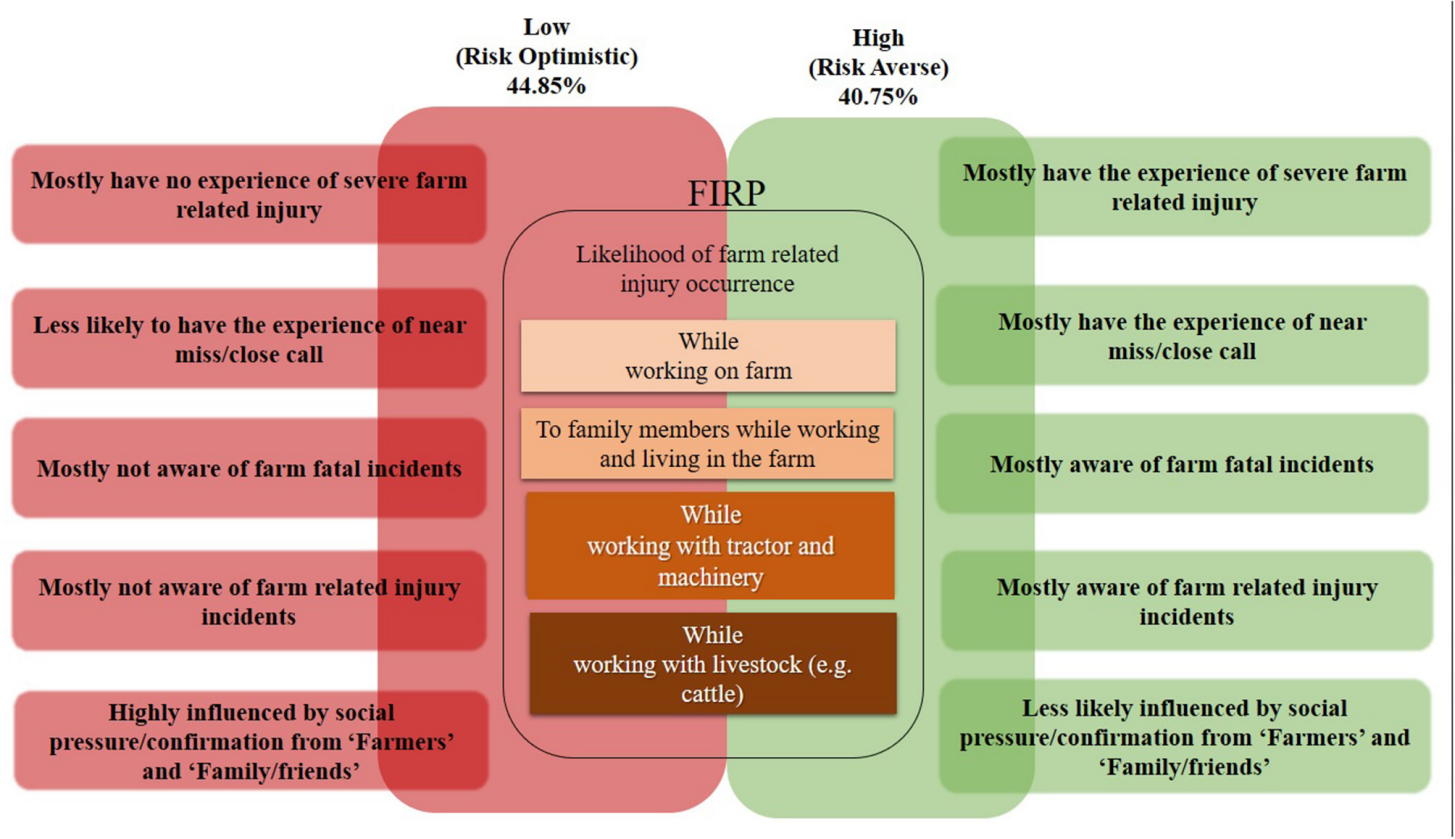
The role of experience, awareness, and social influences on FIRPs.

Accordingly, we conclude that the main reason almost half of the students who participated in this study are “*risk optimistic*” is explained by the fact that they are highly affected by “*farmers*” as key “important others,” and have not experienced a near miss or close call in the past year (Figure 3). This cohort are also less likely to know someone who died in a farm-related incident. The combination of negative social influences of “*family/friends*” on FIRPs along with the lower level of awareness of farm-related incidents contribute to lower FIRPs. When taken in combination, this contributes to their “*optimistic*” perception of risks (Figure 3).

Our findings are in line with the extant research that highlight many younger farmers tend to be “*risk optimistic*” and, consequently, underestimate the risks to themselves and others associated with farming activities (3, 9, 38, 47, 65). Our study showed that over half of the students perceive a lower chance of injury occurrence while working on the farm, with tractor/farm machinery, or livestock. Furthermore, almost the same proportion of students are also “risk optimistic” concerning their family members working/living on the farm. This finding underlines the crucial need for targeting this cohort and improving FIRP through formal education which fosters the development of a “safety mindset” among this cohort as part of the National Safety Plan 2021–2024 (66). To this end, there is a need for tailored educational tools/programs to be embedded in academic education that enhance FIRPs.

Interestingly, our findings show that despite a large group of “*risk optimistic*” students, there is another cohort of students who are “*risk-averse*” and more likely to avoid taking risky actions. This finding is in line with a number of studies which report young farmers may perceive higher levels of risk despite social pressure from their parents (36, 37, 40, 49). Furthermore, comparing the background variables of two cohorts, the study findings illustrate that both “*risk optimistic*” and “*risk averse*” students are almost the same in age, gender proportion, stage of education and, more importantly, general farming experience. Therefore, unlike previous studies that mentioned age, gender and farming experience as significant factors contributing to FIRP (4, 36, 39, 41, 49, 53, 65), we found these factors are not significant in determining whether students are “*risk optimistic*” or “*risk averse*”. Given this finding, it prompts the question of “what leads students with similar socio-demographic characteristics be “*risk optimistic*” and, more importantly, what might influence them to be “*risk averse*”?”

The OLR analysis answers this question and establishes that students form their estimation of the risk of injury occurrence, to themselves or others, based on personal experience of severe injury or a near miss / close call. This finding aligns with studies that found farmers who experienced severe farm injury to be more “*risk-averse*” and less likely to engage in risky actions (4, 9, 28, 43, 47). Young farmers are, however, less likely to be severely injured (16, 32–34); in this study 14% of students reported such incidents. As a consequence, the impact of direct experience is limited with this particular cohort and, consequently, it shows that the majority of young farmers are “*risk optimistic*.”

These findings highlight that students are primarily reactive in terms of shaping their FIRP, i.e., they may enhance FIRPs only after they have experienced a severe injury. In support of this conclusion, we found that, 25% of the students who participated in this study reported their FIRPs being strongly, and positively, affected by a close call or near miss. These results reflect the published literature that highlights that younger farmers mostly experience near misses, close calls or minor injuries (16, 32–34) and adds to this body of work by demonstrating that such experiences result in a positive impact on risk perception, both general and specific. The question then is how to apply this knowledge, i.e. how to provide young farmers with the capacity to assess risk in order to enhance FIRP without them having to experience a severe injury, close call or near miss.

Despite supporting H2c, We found that “farm advisors/university lecturers” have a limited impact on young farmer's FIRP. Whilst the provision of basic knowledge regarding risks and risk assessment are important elements of farm safety education and training, our findings demonstrate that social influences play a stronger role in shaping risk perception. This reflects a growing body of literature that highlights risk-taking behaviors are socially accepted and an established part of farming cultures (13, 14, 19, 20, 49). The findings presented in this paper are in line with a number of studies that found young farmers accrue cultural capital as “authentic farmers” with “important others” by modeling their behaviors on those of older or more experienced farmers (67). It is important to note that this behavior also applies to women and consideration should be given to exploring the underlying motivations of risky behaviors as part of farm safety education or training courses (19). The research presented in this paper highlights the negative influence that “farmers,” in particular, and “family/friends” have on FIRP. The (partial) answer to the question posed at the end of the previous paragraph is to implement blended approaches to learning that simulating the consequences of being “*risk optimistic*.” This can be achieved by incorporating experience sharing of close calls/near misses between young farmers (13, 31), inclusion of survivor testimonials from “leading farmers” within modules or courses (36, 44), inclusion of safety topics within farm visits and group and individual assessment of incident cases using established occupational safety approaches, e.g., Fault Tree Analysis. Education tools should reflect real-life experiences and make tangible the consequences of risky practices or actions for young farmers rather than only focusing on identification of dangers and risks. By grounding educational approaches in practice and moderating the negative impact of existing farming culture *via* social influences of “key important others” it is possible to challenge the social values/norms within farm households and farming communities. This, in turn, can contribute to the reshaping of farm safety culture where being “*risk averse*” is socially valued and preventing risks is deemed to be what a good farmer is expected to do.

## 5. Limitations

Key limitations associated with this research related to the sourcing of the sample from one university in Ireland. UCD has the largest intake of undergraduate students studying agriculture of all the third level education institutions in Ireland. This is a diverse population drawn from all parts of Ireland and includes those from larger, more intensive farm systems where safety incidents are more common and those from smaller or extensive farms. As a consequence of targeting the population of first and second year students, there is insufficient variation in the ages of respondents, ranging 18–20, to test the effect of age on each of the hypotheses developed in this paper. This highlights the need for further research with a broader population, including those in high school or who have recently completed their education and those who are not students but who work/live on farms. Such research would give a better understanding of whether FIRP changes with age and the extent to which the effect of social influences changes with age.

The result that there is a sizable group that are risk optimistic points to the need for additional research to explore the basis for such optimism and associated attitudes and behaviors. The lower levels of experiencing an accident (or at least admitting to it) and awareness of injuries or fatalities may reflects a culture denial, i.e. of not talking about these events. This suggests a need for further research to assess if some students are more open to reporting personal experiences. Equally, research is required to understand whether there are students that are averse to discussing experience of injuries or experience of fatalities. The inclusion of attitudinal questions would be useful in measuring willingness to discuss/report farm safety issues.

Due to the nature of the cross-sectional studies, there are a number of limitations regarding the results, particularly the fact they provide a snapshot of a particular point in time and difficulties in making causal inferences. We sought to overcome the latter limitation by recruiting a large sample population who were, in terms of age, relatively homogeneous. This limits some of the variation that would, otherwise, influence the results. In terms of the snapshot effect, this points to the need for a longitudinal study which would have the benefit of assessing changes in FIRP over time, i.e., measuring if there is a waning in the influence of experience of an injury or knowledge of injuries and death.

Finally, whilst most farms in Ireland can be classified as family farms, i.e., owned and largely operated using family labor and, consequently, are reflective of many farms in other jurisdictions, they have a particular socio-ecological context that may differ substantially compared to other countries.

## 6. Conclusion

This study supports the design and development of FHS modules for young farmers by identifying the key determinants of FIRPs in countries with family farms. According to the findings, to achieve a “safety mindset” among this cohort, the academic institutions should design specific FHS modules underpinned by a community-based co-design approach. This approach can inform curriculum design, education tools, and delivery. This study argues that knowledge exchange and sharing amongst students of first-hand experiences of close calls, near misses, or severe injury and involving guests who are willing to share stories of those who were injured/died in farm incidents will positively impact students' FIRPs. This is important as many young farmers are highly negatively influenced by farmers and family/friends resulting in them underestimating the risks associated with farming. This paper shows that there is a valuable source of knowledge amongst young farmers who have experienced an injury, near miss/call or know someone who died or was injured. This presents an opportunity to include peer knowledge and experiences, provide insights into the context to the incident and show how it has influenced their approach to safety. In developing these approaches there is a requirement to take into consideration the ethical implications and potential consequences of asking young people to publically share knowledge or experiences of traumatic events.

## Data availability statement

The original contributions presented in the study are included in the article/supplementary material, further inquiries can be directed to the corresponding author (david.meredith@teagasc.ie).

## Ethics statement

Ethical review and approval was not required for the study on human participants in accordance with the local legislation and institutional requirements. The patients/participants provided their written informed consent to participate in this study.

## Author contributions

MM and DM developed the conceptual framework and associated research questions addressed in the paper and drafted the questionnaire whilst JM and SF provided expert input to the wording of questions and response options. JM organized the collection of pilot data which were analyzed by MM. SF organized collection of the survey data which were analyzed by MM. MM prepared the initial draft of this manuscript. JM, SF, and JK reviewed the paper prior to submission and provided additional input to the discussion and recommendations. MM and DM reviewed and edited the manuscript. All authors contributed to the article and approved the submitted version.
